# COVID-19 IDD: A global survey exploring family members’ and paid staff’s perceptions of the impact of COVID-19 on individuals with intellectual and developmental disabilities and their caregivers.

**DOI:** 10.12688/hrbopenres.13077.2

**Published:** 2020-12-03

**Authors:** Christine Linehan, Tal Araten-Bergam, Jennifer Baumbusch, Julie Beadle-Brown, Christine Bigby, Gail Birkbeck, Valerie Bradley, Michael Brown, Femmianne Bredewold, Masauso Chirwa, Jialiang Cui, Marta Godoy Gimenez, Tiziano Gomiero, Sarka Kanova, Thilo Kroll, Mac MacLachlan, Brigit Mirfin-Veitch, Jayanthi Narayan, Finiki Nearchou, Adam Nolan, Mary-Ann O'Donovan, Flavia H. Santos, Jan Siska, Tim Stainton, Magnus Tideman, Jan Tossebro

**Affiliations:** 1 UCD Centre for Disability Studies, University College Dublin, Belfield, Dublin, Dublin 4, Ireland; 2 Living with Disability Research Centre, School of Allied Health, Human Services & Sport, La Trobe University, Bundoora, Victoria, 3086, Australia; 3 Canadian Institute for Inclusion and Citizenship, University of British Columbia, 2080 West Mall, Vancouver, BC Canada, V6T 1Z2, Canada; 4 Tizard Centre, University of Kent, Canterbury, Kent, CT2 7NZ, UK; 5 Business Information Systems, University College Cork, O'Rahilly Building, Cork, Ireland; 6 Human Services Research Institute, 2336 Massachusetts Ave, Cambridge, MA 02140, USA; 7 School of Nursing and Midwifery, Queen’s University, 97 Lisburn Road, Medical Biology Centre, Belfast, BT9 7BL, UK; 8 University of Humanistic Studies, Kromme Nieuwegracht 29, Utrecht, 3512 HD, The Netherlands; 9 School of Humanities and Social Sciences, Department of Social Work and Sociology, University of Zambia, Great East Road Campus P.O Box 32379, Lusaka, 10101, Zambia; 10 Department of Social Work, The Chinese University of Hong Kong, Shatin, New Territories, Hong Kong, China; 11 Department of Psychology, University of Almeria, La Cañada de San Urbano, 04120, Almería, Spain; 12 ANFFAS Trentino Onlus DAD© Project Group, via Giambattista Unterveger, 38121 Trento Trentino,, Italy; 13 Department of Education, University of West Bohemia, Univerzitní 2732/8, Plzeň 3, 301 00, Czech Republic; 14 School of Nursing, Midwifery and Health Systems, University College Dublin, Belfield, Dublin, 4, Ireland; 15 School of Psychology, Maynooth University, Maynooth, Ireland; 16 Donald Beasley Institute, 248 Cumberland Street, Dunedin Central, Dunedin 9016, New Zealand; 17 Inclusive Education at Faculty of Health, Education and Society, University of Northampton, Northampton, UK; 18 UCD School of Psychology, University College Dublin, Belfield, Dublin, 4, Ireland; 19 Centre for Disability Studies, Sydney Medical School, Faculty of Medicine and Health, University of Sydney, 92-94 Parramatta Rd, Camperdown, Sydney, NSW 2050, Australia; 20 Department of Special Education, Charles University, Magdalény Rettigové 4, Praha 1, 116 39, Czech Republic; 21 Department of Social Sciences, Ersta Sköndal Bräcke University, Box 441, Sköndal, SE-128 06, Sweden; 22 School of Health and Welfare, Halmstad University, Box 823, Halmstad, SE 301 18, Sweden; 23 Department of Social Work, Norwegian University of Science and Technology, Trondheim, NO-7491, Norway

**Keywords:** Caregivers Carers, Coronavirus, COVID-19, Health Disparity, Intellectual and Developmental Disability, Intellectual Disability, Pandemic

## Abstract

**Background**: This protocol outlines research to explore family members’ and paid staff’s perceptions of the impact of COVID-19 on individuals with intellectual and developmental disabilities and their caregivers. Evidence suggests that people with intellectual and developmental disabilities experience disparities in healthcare access and utilisation. This disparity was evident early in the pandemic when discussions arose regarding the potential exclusion of this population to critical care.

**Methods**: An anonymous online survey will be conducted with caregivers, both family members and paid staff, to explore their perceptions of the impact of COVID-19 in terms of demographics, living arrangements, access to services, social distancing, and carer wellbeing. The survey will be developed by the research team, many of whom are experts in intellectual disability within their own jurisdictions. Using back-translation our team will translate the survey for distribution in 18 countries worldwide for international comparison. The survey team have extensive personal and professional networks and will promote the survey widely on social media with the support of local disability and advocacy agencies. Statistical descriptive and comparative analyses will be conducted. Ethical approval has been obtained for this study from University College Dublin’s Human Research Ethics Committee (HS-20-28-Linehan).

**Dissemination**: Study findings will be prepared in a number of formats in order to meet the needs of different audiences. Outputs will include academic papers, lessons learned paper, practice guidelines, reports, infographics and video content. These outputs will be directed to families, frontline and management delivering disability services, national-level policy makers, healthcare quality and delivery authorities, national pandemic organisations and international bodies.

## Introduction

Intellectual and developmental disability is a term, growing in usage, which acknowledges that intellectual disability is often accompanied by other disabilities including, but not limited to, sensory disability, speech and language difficulties, seizures, behavioural disorder, or difficulties with movement ^1^. Intellectual disability is diagnosed as deficits in intellectual functioning, deficits in adaptive behaviour, and onset during the developmental period ^2– 4^. An estimated 1% of the world’s population has intellectual disability with higher proportions living in low income countries ^5^. Using a global population of 7.7 billion ^6^, it can be estimated that approximately 77 million persons worldwide live with intellectual disability, many of whom will present with additional disabilities such as those listed above.

A paradigm shift in models of disability emphasises the critical need for appropriate support to be available to people with intellectual disability. This position advances previous understandings of disability as either a medical condition requiring a ‘treatment’ or ‘cure’ ^7, 8^ or as a social concept requiring attitudinal and environmental change ^9^. Developed by the American Association on Intellectual and Developmental Disabilities, the support needs model argues that good quality outcomes for people with intellectual disabilities are a function of the support they receive, or *‘put another way, if supports were removed, people with ID (intellectual disability) would not be able to function as successfully in typical activities and settings’* ^4^. The COVID-19 pandemic has disrupted access to supports typically received by people with intellectual and developmental disability and has placed additional challenges on mainstream systems to make adjustments to accommodate need. The impact of these challenges has yet to be empirically assessed.

The United Nation’s Convention on the Rights of Persons with Disabilities affirms the right of persons with disabilities to full inclusion and participation in all aspects of life, charging signatories to the Convention with organising, strengthening and extending support services. Addressing the need for full inclusion, global efforts in deinstitutionalisation have resulted in growing numbers of individuals with intellectual and developmental disability being moved from institutional settings to the community to live in their own home, or in small dispersed community housing often owned by agents such as disability services or mainstream social services ^10, 11^. Paid staff provide various levels of support from drop-in to 24/7. Across all settings, including the family home, there is evidence that appropriate support, drawing on practices such as Active Support, are necessary to promote good quality of life outcomes ^12, 13^. Caregivers therefore play a critical role in the current pandemic. Among many concerns are the impact of caregivers contracting the virus, the challenge of ensuring continuity of care for those who live in community settings, and the situations of those who live and work within large institutional settings.

Article 25 of the Convention specifically affirms the right of persons with disabilities to enjoy the highest attainable standard of health without discrimination on the basis of disability. Despite this protection, many individuals with intellectual and developmental disability experience significant disparities in the prevalence of adverse health conditions and behavioural disorder, attention to healthcare needs, preventative care/health promotion activities, and access to health care ^14^. These disparities were brought into sharp focus by a UK confidential inquiry into mortality which revealed that avoidable deaths from causes amenable to change by good quality healthcare are twice as likely among this population when compared to the general population ^15^. As the COVID-19 pandemic evolves, questions have arisen in the US regarding the legality of specifically withholding COVID-19 treatment from individuals with severe intellectual disability ^16^. Questions have also arisen in the UK where COVID-19 guidelines produced by the National Institute for Health & Care Excellence were deemed to reduce access to critical care for those with intellectual and developmental disabilities, forcing an immediate modification ^17^.

The present study aims to collect survey data on family members’ and paid staff’s perceptions of the impact of COVID-19 on individuals with intellectual and developmental disabilities and their caregivers throughout 18 international jurisdictions. To the authors’ knowledge this will be the first international dataset on this topic.

## Protocol

This research study comprises an international survey of family members and paid staff who provide support to individuals with intellectual and developmental disability. The survey will be conducted by an international network of academics and practitioners. The research team will develop and disseminate an anonymous online survey for completion by family members and paid staff addressing two core questions. Firstly, what is their perception of the impact of COVID-19 pandemic on individuals with intellectual and developmental disability and their caregivers, for example, in access to healthcare and impact of restrictive practices? Secondly, do differences exist in the self-reported experiences of those living in different living arrangements and in different international jurisdictions?

The Principal Investigator is Chair of the Comparative Policy and Practice (CPP) special interest group of IASSIDD, the International Association for the Scientific Study of Intellectual and Developmental Disabilities, the leading professional association in the intellectual and developmental disability field. The CPP membership includes some of the foremost intellectual disability researchers in their respective countries who have previous experience collaborating on research. This group has been supplemented by non-CPP members who bring other expertise to the project including data management, data analysis and translation skills. 

**Figure 1.**
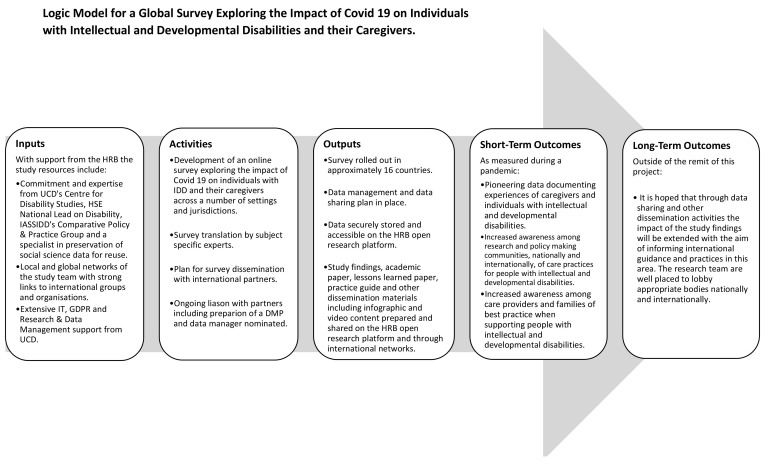
Logic model for coronavirus disease 2019 (COVID-19) intellectual and developmental disabilities (IDD) research project.

To promote clear communication and understanding among the study team, a logic model has been developed outlining the study’s inputs, activities, outputs, short-term outcomes and long-term outcomes. The model provides a useful graphic to concisely encapsulate key areas of the study. The logic model is presented in Figure 1 below.

### Design

This study is a cross-sectional, anonymous, online survey of adult caregivers, comprising family members and paid staff, who support individuals with intellectual and developmental disabilities. This open survey will be hosted online using the platform Qualtrics Core XM ^TM^. The study team, representing 18 countries - Australia, Brazil, Canada, China, Czech Republic, Germany, Greece, India, Ireland, Israel, Italy, Netherlands, Norway, Spain, Sweden, UK, US, and Zambia - will play a key role in creating awareness of the survey in their countries. It is hoped that additional countries may participate if representatives with expertise in this field can be sourced. Additional ethical approval will be sought, as required, for their inclusion.

An advisory group, comprised of a number of study team members, will be established to ensure standardisation in the promotion of the survey. A promotional pack will be developed to include a note outlining the background of study, its purpose and global reach, survey inclusion criteria, ethical approval as well as the study team and funder. A link and a QR code to the survey will also be provided.

### Recruitment of study participants

The participants in this study will self-select to complete the online survey. To facilitate recruitment the study team will engage in a number of activities to promote awareness of the survey and consequently notify caregivers about the survey. Firstly, members of the study team will compile a listing of relevant disability and advocacy organisations within their jurisdiction. The target organisations include those that provide services and support to individuals who have intellectual and developmental disabilities or their family members, ranging from formal state services to informal social media support groups. Promotional information will be shared by the team members in their respective countries and following this, organisations will be invited to disseminate a survey link to staff and more widely through their communication and social media channels. As the study team includes intellectual disability experts in their respective countries, it is anticipated that they can encourage disability and advocacy agencies within their jurisdictions to promote the survey. The study team also anticipates the survey will ‘go viral’ whereby information will snowball beyond the immediate efforts of the team. Similar online surveys of the general population’s experiences during the pandemic have successfully employed this methodology, enjoying high response rates ^18, 19^.

### Sample size calculation

Attempts to determine sample size are challenging. Firstly, to the authors’ knowledge no international online survey of caregivers has previously been undertaken during a pandemic which might provide a reliable estimate. Secondly, in many countries the numbers of persons with intellectual and developmental disabilities and the numbers of caregivers is unknown. Reviews of prevalence estimates of intellectual and developmental disabilities are typically restricted to intellectual disabilities and use administrative rather than population-based data ^20^ Ireland, as the lead country in this research, has data available on the numbers of persons with intellectual disability and caregivers from the national census, which in 2016 were recorded as 66,611 and 195,263 ^21^ respectively, albeit the latter figure is for all caregivers, not just those supporting people who have intellectual and developmental disabilities. Figures for those employed in disability services are equally challenging as, in Ireland, they are reported as Whole Time Equivalent posts rather than individual staff members. To December of 2019, a total of 18,515 Whole Time Equivalent posts were reported working in disability services in Ireland ^22^. Attempting to determine the proportions of caregivers who provide support for persons with intellectual and developmental disabilities, and the actual number of personnel who provide staff support to this population is speculative and therefore has not been determined. Given that sample size calculations are required to determine if sufficient statistical power is available to researchers, it can be argued that if an average of 200 individuals participate in this survey from each of the jurisdictions, the combined sample of 3,200 responses would be more than sufficient to provide the statistical power for any analyses.

### Survey instrument

The anonymous online survey will use only closed quantitative items. While the survey is presented to participants in different languages, the survey software, Qualtrics ^TM^, enables participants to enter their data onto one global dataset. The survey will comprise seven sections, exploring: characteristics of respondents (e.g. gender, age, status of family member or staff); characteristics of person(s) supported (e.g. level of intellectual disability, presence of additional disability, living arrangements); local practices during the pandemic in family home or workplace (e.g. restrictions to typical activity, introduction of new practices, equipment); access to information and training; experience of symptoms, testing, treatment; impact of social distancing; two standardised scales for caregivers measuring mood and the impact of pandemic. Piloting will determine if the format and length of survey is appropriate. Using a process of back translation, the study team will translate the survey into local languages.

### Outcome measures

The primary outcome of interest in this survey is family members’ and paid staff’s perceptions of the impact of COVID-19 on individuals with intellectual and developmental disabilities and their caregivers. The perception of outcomes for people with intellectual and developmental disability are explored generally throughout the survey, but specifically in questions relating to access to health services and protective equipment, continuity of care, adverse impact of restrictions and questions relating to their experiences of symptoms, testing and treatment. The perception of outcomes for caregivers are also explored generally throughout the survey, but specifically in questions relating to mood and impact, using the DASS 12 ^23^ and Coronavirus Anxiety Scale ^24^, and questions relating to their experiences of symptoms, testing and treatment. 

### Data analysis and statistical plan

All data will be analysed using IBM SPSS Version 26 statistical software. Descriptive, comparative, bivariate and multivariate analysis will be conducted to document the circumstances of the respondents and the people they support. Of particular interest are comparative analyses to explore trends by different living arrangement and different jurisdictions.

### Ethics

Ethical approval for the study has been obtained from the host institution University College Dublin [HS 20-28-Linehan]. The study team will each assess the requirement for ethical approval within their own jurisdiction. It is not envisaged that the study team will apply for ethical approval from individual disability agencies as to do so would take considerable time and would likely create a circle of amendments which would collectively need to be agreed by all parties. It is also important to note that this research is an anonymous survey. There is no opportunity for participants to identify either themselves or the organisations they work for or engage with.

The study team acknowledge the sensitive nature of this topic and identified a number of ethical issues to the ethical approval body with actions to respond to each issue. Individuals who have intellectual and developmental disability, or support a person or persons with intellectual and developmental disability, may have experienced adverse effects to social distancing, may have contracted the virus or know of family and/or friends who did, and indeed may have experienced the death of family and/or friends to the virus. Reflecting on their experiences, and the experiences of those they support, it is possible that some participants may become distressed when completing the survey. For this reason, participants are directed to national and/or local support services should they wish to avail of support.

The study team is also cognisant of the fact that participants may be aware of cases of abuse, neglect and/or exploitation during this period. Given the anonymous nature of the survey, and the use of closed items, participants will be unable to detail these experiences to the study team. Instead, participants will be advised to direct any such concerns to the appropriate relevant authorities which will be identified for their attention.

The study team acknowledge that the study does not involve people with intellectual and developmental disabilities in its design or collection of data instead relying on proxy perspectives and experiences of family members and paid staff. This is a limiting factor as critical experiences and views will be missing from the findings. This study was funded as a ‘rapid’ grant at a time when many of the researchers were in lockdown and many disability services were severely restricted in the services they could provide, notably as their focus was on reducing risk of infection and maintaining high quality support, both of which were impacted by staff absences caused by the need to self-isolate. A pragmatic decision was required as to what methodology was feasible and an online and anonymous survey of caregivers was deemed most appropriate. It is the opinion of the researchers, that typical methodologies which include the direct participation of individuals with intellectual and developmental disabilities, such as interviews and focus groups, were not feasible options at this time. The availability of the various types of individualized support usually provided by advocates, self-advocacy groups and service providers required to enable people with intellectual and developmental disabilities to participate in surveys or other forms data collection were simply unavailable given the pressures created by the pandemic. It could be argued that individuals with low support needs may not have required additional support to complete a survey tailored to their needs. Strict lock down regimes in many countries, however, meant that even among this group many did not have access to or familiarity with digital devices necessary to complete the survey. It is also important to recognize that even in the best of circumstances there will always be a group of people with more severe and profound intellectual disabilities whose direct experiences cannot be ascertained through spoken or other symbolic forms of communication. For this group, reliance on others who know them well, such as family or support workers is often the only way of gaining insights into their experiences. To address this limitation the researchers have engaged with Inclusion International who will support a group of self-advocates, preferably from the participating countries, to guide the interpretation of findings and recommendations. It is also important to note that since emerging from lockdown, a number of the research teams are actively working on studies that will directly capture the lived experience of individuals with intellectual and developmental disabilities during the COVID-19 pandemic, notably in Ireland, the Netherlands and USA. It is hoped that findings from this survey will inform these studies.

Finally, ethical issues arise regarding the security and anonymity of the online survey, notably as participants are informed that the dataset will be uploaded to an open data portal for use by other researchers. To address these issues, the study team will develop an anonymous survey, without collecting IP addresses that may provide a link to participation, and will use closed response items to ensure that no communication that may be identifiable can be received from participants. The survey is hosted on the Qualtrics platform which has been successfully used in previous large-scale surveys by the lead institution, University College Dublin in Ireland.

### Data management and dissemination

**Figure 2.**
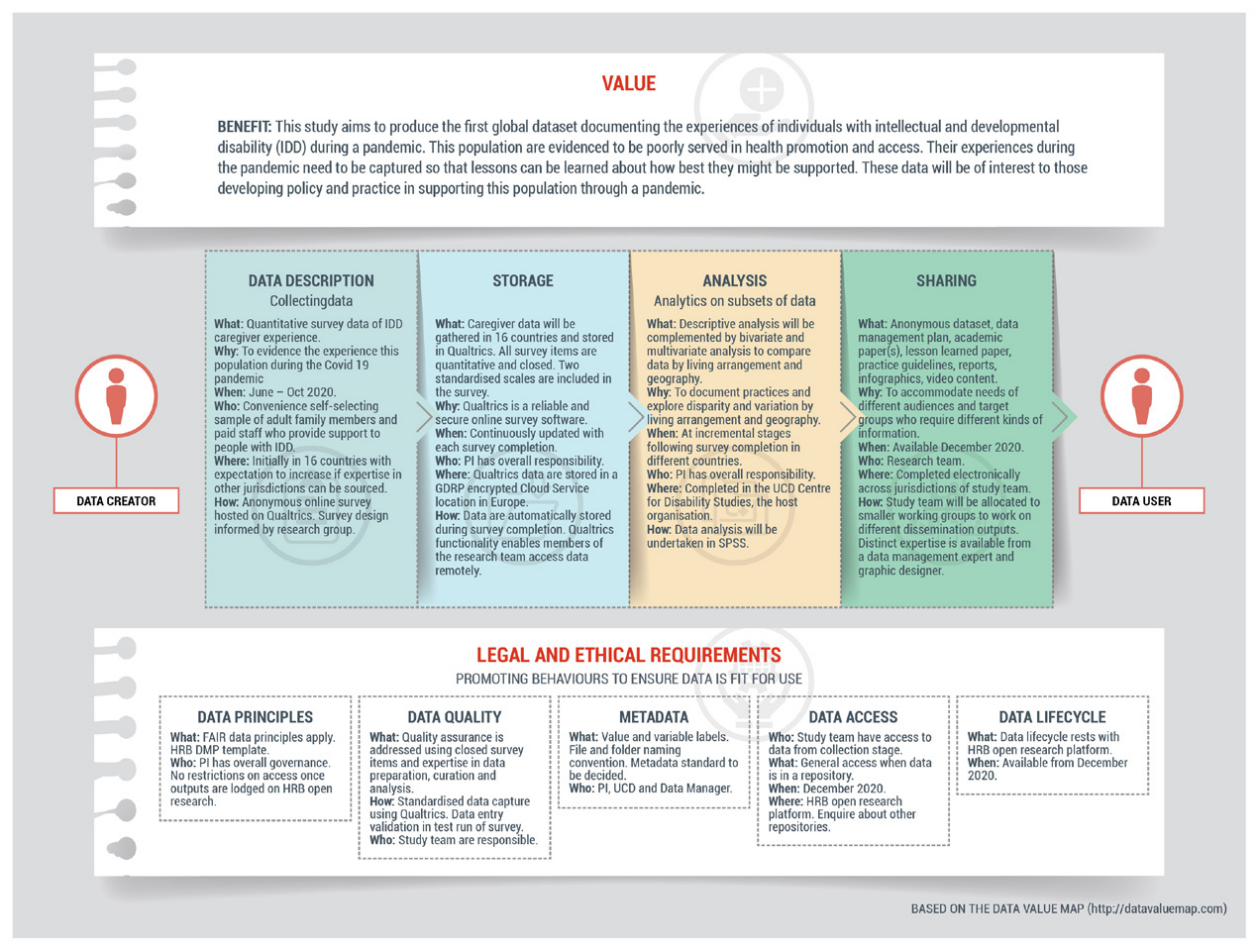
Visualised data management plan for coronavirus disease 2019 (COVID-19) intellectual and developmental disabilities (IDD) research project.

A comprehensive data management plan will be developed by the study team and all data gathered will be shared on an approved data repository. The data management plan is due for publication on the HRB Open Research in Month 3 of the project. In preparation for this, the study team used an adapted version of the Data Value Map ^25^, as a discursive template to facilitate a conversation about the data management requirements for this study. In line with the HRB Data Management Planning template, issues relate to data collection, data storage, data analysis, data sharing and preservation, and ethical and legal requirements, as well as who will be responsible for each stage. This discussion helped to forge an appreciation of the open research lifecycle among the study team and the value that will accrue from this process. A visualisation of this plan, developed by Gail Birkbeck (co-author), is presented in Figure 2.

Study findings will be prepared in a number of formats in order to meet the needs of different audiences. These outputs include academic papers, lessons learned paper, practice guidelines, reports, infographics and video content. These outputs will be directed to the following stakeholders: people with intellectual and developmental disabilities and their families via local advocacy groups; frontline and management delivering disability services; national-level policy makers, healthcare quality and delivery authorities; national pandemic organisations (e.g. the National Public Health Emergency Team, the Health Protection Surveillance Centre); and international bodies responding to the pandemic such as WHO, Fundamental Rights Agency, Amnesty, and EASPD (European Association of Service Providers for Persons with Disabilities). The research team, via IASSIDD and their own personal contacts, also have significant networks to realise extensive dissemination, knowledge exchange and data sharing of the findings and learnings from this survey. While the lead investigator will take responsibility for global dissemination, country leads will be encouraged to undertake dissemination for their own jurisdiction.

When completed, this study will be reported using CHERRIES: Eysenbach G. Improving the quality of Web surveys: the Checklist for Reporting Results of Internet E-Surveys (CHERRIES). J Med Internet Res. 2004; 6(3):e34.

### Study status

This study has not yet commenced data collection.

## Conclusion

Despite the protections of the UN Convention on the Rights of Persons with Disability, people with intellectual and developmental disabilities are at risk of health disparities when compared with the general population. This study aims to gather international data on the experiences of individuals with intellectual and developmental disabilities and their caregivers during the COVID-19 pandemic. These data will provide a first glimpse of the challenges which arose for this population and their caregivers during the pandemic. Of particular interest is whether experiences varied by living arrangement and by country, and whether lessons can be learned to inform policy and practice for future pandemics. By depositing the anonymous dataset on an open forum, other researchers are encouraged to continue the exploration of these data.

## Data availability

### Underlying data

No data are associated with this article.
