# Supplementary material for: COVID-19 IDD: A global survey exploring family members’ and paid staff’s perceptions of the impact of COVID-19 on individuals with intellectual and developmental disabilities and their caregivers
Source: HRB Open Res. 2020 Dec 3;3:39. Originally published 2020 Jun 23. [Version 2] doi: 10.12688/hrbopenres.13077.2 (PMC7745183; doi:10.12688/hrbopenres.13077.2)
Supplement: Peer review history

# Peer review: Reviewer response for version 2

**DOI:** 10.21956/hrbopenres.14289.r28498

**Published:** 2020-12-09

**Authors:** Germain Weber

**Affiliations:** 1 Department of Clinical and Health Psychology, Faculty of Psychology, University of Vienna, Vienna, Austria

The revised version is fine with me, as authors have clearly switched their focus and study aim to the perception of family members and front-line staff on COVID19 related effects for people with IDD! Further authors elaborate on a pragmatic justification for not having included the direct voice of people with IDD in this survey.

 Our earlier recommendation to make use of effect values and not p-values in data analysis remains.

 With regard to the section “Sample size calculation”, expected responses of 200 per jurisdiction would come up to 3.600 as the revised version includes 16 countries!

Is the study design appropriate for the research question?

Partly

Is the rationale for, and objectives of, the study clearly described?

Partly

Are sufficient details of the methods provided to allow replication by others?

Yes

Are the datasets clearly presented in a useable and accessible format?

Not applicable

Reviewer Expertise:

NA

I confirm that I have read this submission and believe that I have an appropriate level of expertise to confirm that it is of an acceptable scientific standard.

# Peer review: Reviewer response for version 1

**DOI:** 10.21956/hrbopenres.14176.r27914

**Published:** 2020-09-16

**Authors:** Germain Weber, Elisabeth Zeilinger

**Affiliations:** 1 Department of Clinical and Health Psychology, Faculty of Psychology, University of Vienna, Vienna, Austria; 2 Faculty of Psychology, University of Vienna, Vienna, Austria

The research protocol as described is well planned and organized. The topic is of eminent relevance with regard to health provision and equal opportunities for people with intellectual disability in a period of pandemic. Though the authors do not mention it, the research as planned refers directly to article 11 of the UN-CRPD.

 Having said this, we have to address one major concern and several additional issues listed below:

 Major concern:

- Authors state that the main goal of the study is to assess the **experience and the impact** of the pandemic **of and on** **persons with ID** and their caregivers. However, persons with ID are not directly included in the study. We highly recommend the consortium to respect the requirements of the UN-CRPD and adhere to the principle “nothing about us without us”. We agree that it will take special effort to include persons with ID as direct participants in the study. However, the pandemic is not a good reason to exclude persons with ID from the study. Without the direct experience as expressed by persons with ID the study findings will be of limited impact. We therefor demand the consortium for a change and include people with ID in an acceptable way in the sample. In countries like the UK, Ireland, Australia, the Netherlands, Norway and France with substantial advances and supports for self-advocates this inclusion is more than realistic!

Further aspects:

1. We suggest to reflect on sample representativeness in a differentiate way. Even if in most countries, there is only fragmented knowledge about the characteristics of the sample, data collection should aim at some kind of representativeness. Otherwise, results cannot be generalised, and their full potential will remain unfolded. A representative sample might be randomly selected from a larger dataset after data collection, resulting in a stratified sample. Further, authors are not quite consistent when offering general epidemiological data on people with ID data in the first section of the introduction, and stating later in the section of sample size that the number of people with ID remains unknown in many countries.
2. Considering the statistical analysis, the fact that the total sample in this study will be far beyond 1,000 persons has to be addressed in the study protocol. We highly recommend authors not to use p-values for any interpretation. Using p-values for interpreting results is never advisable with this sample size though unfortunately still frequently done. With such a large sample size, every test will be “statistically significant” with p-values < 0.01. However, this would be an artefact resulting from the large samples size. Thus, we strongly recommend making use of effect sizes for interpretation, as they are independent of sample size.
3. We have some concerns with using only closed questions for data collection. The study design has the potential to explore all kinds of matters arising in relation to the pandemic, even aspects that may not have been on the authors’ minds. Thus, including open-ended questions will provide the opportunity to address aspects not specifically selected primary to data collection.
4. Finally, we advise authors to go more into detail with dissemination. The impact of the study will be highly dependent on a dissemination plan including various stakeholders. Many dissemination frameworks available can guide dissemination activities.

Thank you for offering us the opportunity to review this study protocol. May the consortium understand our concerns and recommendation in the way to transform a good study protocol in an excellent one!

Is the study design appropriate for the research question?

Partly

Is the rationale for, and objectives of, the study clearly described?

Partly

Are sufficient details of the methods provided to allow replication by others?

Yes

Are the datasets clearly presented in a useable and accessible format?

Not applicable

Reviewer Expertise:

Psychology and intellectual Disability. Health promotion and disability. Disability and inclusive research.

We confirm that we have read this submission and believe that we have an appropriate level of expertise to confirm that it is of an acceptable scientific standard, however we have significant reservations, as outlined above.

# Response

**Published:** 2020-10-02

**Authors:** Christine Linehan

**Affiliations:** University College Dublin, Ireland

(1) We thank Reviewers #2 and 3# for their combined review, and for describing the study as being of eminent relevance. We note that through no fault of their own, this review was received when data collection had just one week to run. While the benefits of open publishing of study protocols are many, one challenge is the possibility that rapid research may be near to completion by the time reviews are received. The reviewers may notice that we have added authors who were recruited to gather data in additional countries during this time period.

(2) We understand the reviewers’ major concern regarding the fact that individuals with intellectual and developmental disabilities were not invited to complete the survey. We agree with the principle of ‘nothing about us without us’, however the pandemic did impact on our choice of methodology. This study was funded as a ‘rapid’ grant at a time when many of the researchers were in lockdown and many disability services were severely restricted in the services they could provide, including supports to advocacy groups which we agree are present in some of the participating countries. Furthermore, in some countries at the time of the survey, the attention of many service providers was totally focused on reducing risk of infection to people with intellectual and developmental disabilities in their services and maintaining high quality support, and were experiencing extreme pressure in doing so by staff absences caused by the need to self-isolate.

A pragmatic decision was required as to what methodology was feasible and an online and anonymous survey of caregivers was deemed most appropriate. It is the opinion of the researchers, that typical methodologies which include the direct participation of individuals with intellectual and developmental disabilities, such as interviews and focus groups, were not feasible options at this time. The availability of the various types of individualized support usually provided by advocates, self-advocacy groups and service providers required to enable people with IDD to participate in surveys or other forms data collection were simply unavailable given the pressures created by the pandemic.  

It could be argued that individuals with low support needs may not have required additional support to complete a survey tailored to their needs. Strict lock down regimes in many countries, however, meant that even among this group many did not have access to or familiarity with digital devices necessary to complete the survey. It is also important to recognize that even in the best of circumstances there will always be a group of people with more severe and profound intellectual disabilities whose direct experiences cannot be ascertained through spoken or other symbolic forms of communication. For this group, reliance on others who know them well, family or support workers is often the only way of gaining insights into their experiences.

We have made the following revisions to address this issue. We have revised the title of the study to highlight that it is caregivers’ perceptions which are captured.

The original title was:

COVID-19 IDD: A global survey exploring the impact of COVID-19 on individuals with intellectual and developmental disabilities and their caregivers. The new title is:

COVID-19 IDD: A global survey exploring family members’ and paid staff’s perceptions of the impact of COVID-19 on individuals with intellectual and developmental disabilities and their caregivers.

We have also revised our protocol to include the fact that our findings will be reviewed in a consultation with an international forum of self-advocates from participating countries, an arrangement that was not formalized at the time the study protocol was published. Finally, it is important to note that a number of partners are conducting qualitative studies involving individuals with intellectual and developmental disabilities to give voice to their experiences during the pandemic, specifically these studies are planned in Ireland and in the US. These opportunities were not possible in the early stages of the pandemic when this study was planned. We hope the findings of this study will inform these studies.

(3) As noted to our first reviewer, to address the issue of representativeness, where national data permits, we will weight the samples by level of intellectual ability (mild, moderate, severe/profound). Where national data is not available, we will weight the sample by international distributions of level of ability. We have revised the apparent contradiction where we present general epidemiological data yet state that the number of persons with intellectual and developmental disabilities remains unknown in many countries.  A recent review of attempts to estimate the prevalence of IDD (Friedman et al, 2018) specifically excluded the term ‘developmental disabilities’ in favour of focusing on intellectual disabilities doi: 10.1111/jppi.12220.  

(4) Where inferential statistics are conducted, we will present both p values and effect sizes which we agree is a transparent and appropriate reporting of difference.

(5) Where inferential statistics are conducted, we will present both p values and effect sizes which we agree is a transparent and appropriate reporting of difference.

Regarding the proposal to include qualitative questions on the survey, regrettably the reviewers’ commentary was received with just one week remaining in data collection and we are therefore unable to change the survey. The rationale for closed questions was to facilitate back-translations of the survey into 15 languages and to avoid having another series of translations of respondents’ qualitative data. The survey is substantive, taking 20-25 minutes to complete, and resources were scare with funding of just one research assistant. Finally, the researchers also wanted to ensure that no opportunities arose for respondents to threaten the anonymous nature of the survey through qualitative comments.

(6) We agree that a dissemination plan would be helpful. Our plan is that in each country lead researchers will be take responsibility for writing results into more accessible formats to complement the academic outputs, and where possible will assist in disseminating results to local governments, advocacy groups, and other interested stakeholders using the similar range of media to that used for recruiting participants.

(7) We thank Reviewers #2 and #3 for their helpful suggestions to improve the quality of this study.

# Peer review: Reviewer response for version 1

**DOI:** 10.21956/hrbopenres.14176.r27575

**Published:** 2020-07-14

**Authors:** Gloria L. Krahn

**Affiliations:** 1 College of Public Health and Human Sciences, Oregon State University, Corvallis, OR, USA

The proposed study is critically important and timely; the authors/survey planners are commended for their speed in developing an approach to assess the impact of COVID-19 on a population that often gets forgotten. The article is well-written, incorporates many of the concerns that are being voiced now, and proposes to generate data that will provide valuable information. 

 Questions re: Appropriateness of Design relate to:

1. What efforts will be made to reflect any degree of representativeness in the sample (e.g., comparison with what is known about persons with IDD in each country)? Without some understanding of representativeness, how will findings be interpreted, and interpreted across countries? Will any options be available for responses from persons who do not have internet access?
2. The respondents will necessarily be those who have not become ill or very ill or died due to COVID-19. Will any data be available on hospitalizations or deaths to complement the survey findings?
3. The issue of only carer responses is a concern, as noted by the authors. Was thought given to supplementing with case studies with persons with IDD to capture some of their expressed experiences?

Questions re: Sufficient Details relate to the survey questionnaire still under development.

 Because this is a prospective proposal, several comments are added for consideration:

1. Potential impact - in addition to the outcomes to be measured, will there be any questions related to value of training or materials for persons with IDD and carers in learning how to understand and cope with COVID.
2. Any other opportunities for collecting information on “valuable practices”?
3. The public health, medical, and general communities are learning so much about the disease as we gain more experience with it. Will information for each country be “time-stamped” in some way (e.g., date? Or time relative to when the virus became more prevalent in their country?)
4. What information will be collected about their current situation, such as access to clean water in low and middle income countries, or opportunities for social distancing in densely populated urban settings?

Thank you for the opportunity to comment.

Is the study design appropriate for the research question?

Partly

Is the rationale for, and objectives of, the study clearly described?

Yes

Are sufficient details of the methods provided to allow replication by others?

Partly

Are the datasets clearly presented in a useable and accessible format?

Not applicable

Reviewer Expertise:

Disability and public health; disability epidemiology and policy; data for health equity and IDD.

I confirm that I have read this submission and believe that I have an appropriate level of expertise to confirm that it is of an acceptable scientific standard.

# Response

**Published:** 2020-10-02

**Authors:** Christine Linehan

**Affiliations:** University College Dublin, Ireland

(1) We thank the Reviewer #1 for endorsing the critical importance of this study. We would like to inform the reviewer that we were asked to hold on a response until a second review was posted over two months later, hence the delay in responding.

(2) To address the issue of representativeness, where national data permits, we aim to weight the samples by level of intellectual ability (mild, moderate, severe/profound). Where national data is not available, we will weight the sample by international distributions of level of ability.

Only those with internet access can complete the survey, typically on smart phones, tablets or computers. Due to the length of the survey, and the complex branching logic for different respondents, it was not possible to circulate paper copies. Due to social distancing it was not possible for the researchers to lend direct support in completing the survey.

(3) We acknowledge that those who have died from COVID 19 are excluded and that those who are ill or very ill at the time of the survey will be likely excluded or underrepresented. We are hopeful that those who were previously ill or very ill will take part as we ask respondents about their own experience of contracting COVID-19, including possible hospitalisations. We also include questions as to whether the person(s) with intellectual and developmental disability respondents support contracted the virus, were hospitalized, and survived COVID-19.

(4) We agree the issue of caregiver-only responses is a concern. We refer the reviewer to our response below to Reviewers #2 and #3 who also raised this issue.

(5) Regarding the comments for consideration, we agree there is an opportunity to explore potential impact. We included questions on the availability and quality of training and easy to read materials, and we included standardised measures of caregiver mood and anxiety of COVID-19.

(6) In our forthcoming paper reporting on findings, we will ‘time stamp’ each country by presenting information such as the total number of deaths and current cases in each country at the time of data collection.

(7) Regarding data on participants’ current situation, we did not ask about access to clean water or density of population within regions, rather we addressed current situation by including questions on possible reorganization of staff shifts to reduce contacts, reduction in contact with family and friends, use of communications such as phone, internet to keep in touch etc.

(8) We thank the reviewer for taking the time to review this paper and offering some very useful insights to improve the study.
